# Correction to: Late Neogene evolution of the Peruvian margin and its ecosystems: a synthesis from the Sacaco record

**DOI:** 10.1007/s00531-021-02126-5

**Published:** 2021-12-10

**Authors:** Diana Ochoa, Rodolfo Salas-Gismondi, Thomas J. DeVries, Patrice Baby, Christian de Muizon, Alí Altamirano, Angel Barbosa-Espitia, David A. Foster, Kelly Quispe, Jorge Cardich, Dimitri Gutiérrez, Alexander Perez, Juan Valqui, Mario Urbina, Matthieu Carré

**Affiliations:** 1 Laboratorios de Investigación y Desarrollo (LID), Centro de Investigación Para el Desarrollo Integral y Sostenible (CIDIS), Facultad de Ciencias y Filosofía, Universidad Peruana Cayetano Heredia, Av. Honorio Delgado 430, Lima, Peru; 2 Departamento de Paleontología de Vertebrados, Museo de Historia Natural, Universidad Nacional Mayor de San Marcos, Lima, Peru; 3 Burke Museum of Natural History and Culture, University of Washington, Seattle, WA 98195 USA; 4 Géosciences-Environnement Toulouse, UMR CNRS/IRD/Université Paul Sabatier, 14 Avenue Edouard Belin, 31400 Toulouse, France; 5 CR2P (CNRSMNHN, Sorbonne Université), Département Origines et Évolution, Muséum national d’Histoire naturelle, case postale 38, 57 rue Cuvier, 75231 Paris Cedex 05, France; 6 Department of Geological Sciences, University of Florida, 241 Williamson Hall, Gainesville, FL 32611 USA; 7 Instituto de Investigaciones en Estratigrafía (IIES), Universidad de Caldas, Calle 65 # 26-10, edificio Orlado Sierra, Bloque B, segundo piso, Manizales, Colombia; 8 Programa de Maestría en Ciencias del Mar, Universidad Peruana Cayetano Heredia, Lima, Peru; 9 Instituto del Mar del Peru (IMARPE), Dirección de Investigaciones Oceanográficas, Callao, Peru; 10 LOCEAN Laboratory, Sorbonne Université (UPMC)-CNRS-IRD-MNHN, Paris, France

## Correction to: International Journal of Earth Sciences (2021) 110:995–1025 10.1007/s00531-021-02003-1

The article “Late Neogene evolution of the Peruvian margin and its ecosystems: a synthesis from the Sacaco record” written by Diana Ochoa, Rodolfo Salas-Gismondi, Thomas J. DeVries, Patrice Baby, Christian de Muizon, Alí Altamirano, Angel Barbosa-Espitia, David A. Foster, Kelly Quispe, Jorge Cardich, Dimitri Gutiérrez, Alexander Perez, Juan Valqui, Mario Urbina and Matthieu Carré was originally published Online First without Open Access. After publication in volume 110, issue 3, pages (995–1025), the author decided to opt for Open Choice and to make the article an Open Access publication. Therefore, the copyright of the article has been changed to © The Author(s) 2020 and the article is forthwith distributed under the terms of the Creative Commons Attribution 4.0 International License, which permits use, sharing, adaptation, distribution and reproduction in any medium or format, as long as you give appropriate credit to the original author(s) and the source, provide a link to the Creative Commons licence, and indicate if changes were made. The images or other third party material in this article are included in the article’s Creative Commons licence, unless indicated otherwise in a credit line to the material. If material is not included in the article’s Creative Commons licence and your intended use is not permitted by statutory regulation or exceeds the permitted use, you will need to obtain permission directly from the copyright holder. To view a copy of this licence, visit http://creativecommons.org/licenses/by/4.0.

The original article has been corrected.

